# Letter to the Editor: Economic Burden of Type 2 Diabetes Mellitus Among Patients Attending Ho Teaching Hospital in the Ho Municipality, Ghana: A Cross‐Sectional Study

**DOI:** 10.1002/hsr2.72969

**Published:** 2026-08-02

**Authors:** Muhammad Mehr Ali, Sandesh Kumar Manglani, Aazaz Ahmad Khalid

**Affiliations:** ^1^ Northwest School of Medicine Peshawar Pakistan; ^2^ Khairpur Medical College Khairpur Mirs Pakistan

## Author Contributions

Muhammad Mehr Ali has done conceptualization, methodology, project administration, writing – original draft, writing – review and editing. Sandesh Kumar Manglani has done methodology, validation, writing – original draft. Aazaz Ahmad Khalid has done methodology, writing – original draft, writing – review and editing.

## Funding

The authors have nothing to report.

## Disclosure

The authors declare that they have no financial relationships, affiliations, or potential conflicts of interest with any organization or entity that could be perceived as having an interest in the subject matter of this letter.

## Ethics Statement

The authors declare that this manuscript has been prepared in accordance with Wiley's Best Practice Guidelines on Publishing Ethics and the principles of the Committee on Publication Ethics (COPE). The work has been conducted ethically and responsibly, without research misconduct including data fabrication, data falsification, plagiarism, duplicate publication, inappropriate authorship practices, or undeclared conflicts of interest. All authors have reviewed and approved the final manuscript and agree to be accountable for all aspects of the work.

## Consent

All authors have read and approved the final version of the manuscript Muhammad Mehr Ali had full access to all of the data in this study and takes complete responsibility for the integrity of the data and the accuracy of the data analysis.

## Conflicts of Interest

The authors declare no conflicts of interest.

## Transparency Statement

The Muhammad Mehr Ali affirms that this manuscript is an honest, accurate, and transparent account of the study being reported; that no important aspects of the study have been omitted; and that any discrepancies from the study as planned (and, if relevant, registered) have been explained.

Editor,

We read with great interest the recently published study by Dalaba et al. (2026), “Economic Burden of Type 2 Diabetes Mellitus Among Patients Attending Ho Teaching Hospital in the Ho Municipality, Ghana: A Cross‐Sectional Study.” [1] The authors deserve praise for tackling a significant and little‐studied aspect of diabetes care in sub‐Saharan Africa and producing useful empirical data on the direct and indirect expenses that patients at the tertiary level must deal with. Despite nearly universal registration in Ghana's National Health Insurance Scheme, the discovery that almost all patients faced catastrophic medical expenses is startling and pertinent to policy. However, we would like to draw attention to a number of methodological issues that could compromise the reliability and applicability of the published results.

Initially, only patients from one tertiary referral center were included in the study. For comparable contexts, Ho Teaching Hospital handles the most complicated, chronic, and complication‐prone cases, which are by nature more costly to handle than district hospitals. Therefore, rather than representing the national average, the stated mean monthly cost of USD 21.25 and the catastrophic health spending prevalence of 100% at the 10% income barrier probably indicate the may reflect higher‐end estimates. When opposed to single tertiary‐center research, multi‐level facility designs—sampling patients concurrently across primary, district, and tertiary care—have been demonstrated to produce much lower cost estimates. This contextual gap restricts the current findings’ relevance to policy [2].

Second, for comparable context independent of each participant's actual income, household income was uniformly proxied for all 118 patients using Ghana's 2024 national minimum wage (USD 36.30). Because the study's own sociodemographic data reveals significant occupational variability, such as public servants (29.66%) and pensioners (16.10%), who probably make significantly more than the minimum wage, this is an important methodological consideration. Catastrophic health expenditure rates are mathematically inflated when a single low‐income proxy is applied to all participants, especially at the 10% threshold. Population‐level income proxies introduce consistent upward bias in catastrophic health spending calculation, particularly in communities with varied employment, as Wagstaff and Van Doorslaer have officially shown. This issue is particularly relevant in this context [3]. Future studies should collect actual income through validated range‐response categories or supplementary asset‐based wealth indices to stratify catastrophic health expenditure findings by true socioeconomic status.

Third, the cross‐sectional design records expenses at a single moment in time, namely, patient‐recalled spending from the previous month, and then extrapolates this to an annual total of USD 255.05. However, the expenses associated with managing diabetes vary significantly over the course of a patient's illness, peaking during acute problems, hospital stays, or increased treatment intensity, and stabilizing during times of controlled glycemia. This temporal fluctuation cannot be captured by a 1‐month cost frame. According to Kirigia et al., cross‐sectional single‐point cost estimates for diabetes in African settings may substantially underestimate genuine annual costs because of missing hospitalization episodes and sporadic expenditure spikes caused by complications [4]. Prospective longitudinal follow‐up with quarterly cost assessments, or retrospective 12‐month medical record review, would substantially improve the accuracy of annual cost estimates.

Fourth, the study's cost methodology ignored expenses associated with comorbid illnesses and only included diabetes‐specific expenses. Type 2 diabetes most frequently coexists with hypertension, dyslipidemia, and early chronic kidney disease in Ghana and throughout sub‐Saharan Africa; each of these conditions necessitates additional medication and laboratory monitoring. The stated monthly number of USD 21.25 is probably a significant underestimation of the overall health spending burden experienced by these individuals due to the absence of these comorbidity‐driven costs. The 75th percentile of monthly direct care expenditures increased to USD 118.88 when type 2 diabetes and hypertension comorbidity were taken into account, as shown by Amon et al. in Ghana. This is significantly higher than what a diabetes‐only framework would capture [5]. To prevent systematic underestimating of the actual economic burden, future cost‐of‐illness studies in diabetes populations should use a comorbidity‐inclusive expenditure approach.

In conclusion, aligning with our aim to critically evaluate the study's methodological framework, we acknowledge that Dalaba et al. provide a valuable contribution regarding diabetes‐related financial hardship in Ghana. However, our evaluation indicates that the interpretation of the reported economic estimates is heavily influenced by the reliance on a single tertiary facility, the application of a uniform minimum‐wage income proxy, the use of a cross‐sectional single‐month cost recall, and the omission of comorbidity‐driven expenses. By addressing these specific methodological constraints, future research utilizing multi‐level facility designs, validated income stratification instruments, longitudinal cost tracking, and comorbidity‐inclusive costing frameworks will generate more robust and representative data for health system decision‐making.

## Data Availability

Data sharing is not applicable to this article as no data sets were generated or analyzed during the current study.
